# Challenges in Assessing Combined Interventions to Promote Linear Growth

**DOI:** 10.4269/ajtmh.17-0212

**Published:** 2018-02-12

**Authors:** Emily L. Deichsel, Kirkby D. Tickell, Jessica E. Long, Nelson L. Jumbe, Ali Rowhani-Rahbar, Judd L. Walson

**Affiliations:** 1 Department of Epidemiology, University of Washington, Seattle, Washington;; 2 Department of Global Health, University of Washington, Seattle, Washington;; 3 Childhood Acute Illness & Nutrition Network (CHAIN), Nairobi, Kenya;; 4 Pharmactuarials LLC, Mountain View, California;; 5 Department of Pediatrics, University of Washington, Seattle, Washington;; 6 Department of Medicine, University of Washington, Seattle, Washington

## Abstract

Despite the recognition of stunting as a public health priority, nutritional and nonnutritional interventions to reduce or prevent linear growth failure have demonstrated minimal impact. Investigators and policymakers face several challenges that limit their ability to assess the potential benefits of combining available interventions into a linear growth promotion package. We use two common but very different interventions, deworming and multiple micronutrient supplements, to illustrate barriers to recommending an optimal linear growth promotion package based on the currently available literature. These challenges suggest that combining individual- and population-based as well as model-based approaches would complement existing research using systematic review, meta-analysis, and factorial randomized trials, and help integrate existing fields of research to inform the development of optimal linear growth promotion packages for children living in resource-limited settings.

## INTRODUCTION

More than 165 million children worldwide are stunted (height-for-age *z*-score [HAZ] < −2), a marker of chronic malnutrition.^1^ Stunting is associated with substantial morbidity and mortality, including a 2-fold increase in the risk of death before age 5.^1,2^ Although stunting is a public health priority, current interventions have demonstrated minimal effect in reversing or preventing stunting. Estimates suggest that if available growth-promoting interventions reached 90% of their target population, stunting incidence would only be reduced by one-fifth.^3^ The combination of proximal etiologic causes of stunting, including lack of adequate nutrition, hormonal dysregulation, repeated infections, environmental enteric dysfunction, and chronic systemic inflammation, occurs within a complex network of more distal factors that underlie stunting.^2^ As a result of the multifactorial etiologies of stunting, successful prevention or treatment may require combined packages of interventions targeting multiple pathophysiologic pathways leading to stunting. The failure to prevent or reverse stunting may result from the complex interactions between prenatal and early childhood health insults that result in linear growth failure.

There is increasing interest in determining the effects of combination packages of interventions that may or may not have been evaluated together in clinical trials. Although many randomized trials of potentially growth-promoting interventions have been conducted, interventions are usually not tested in different combinations to establish optimal packages for linear growth promotion. Investigators using currently available literature to evaluate the potential benefit of combining interventions into useful packages to prevent or treat stunting face several methodological challenges that limit their ability to assess the potential combination benefits of available interventions. An understanding of these challenges and the limitations of traditional methods should encourage researchers and policymakers to consider the use of nontraditional methods designed to integrate data and model testing of different intervention combinations.

This article highlights two commonly delivered but very different interventions, deworming and multiple micronutrient supplementation, that are administered concurrently across a range of geographic areas. We use these examples to illustrate challenges in constructing a linear growth promotion package using currently available evidence.

## DEWORMING: INTERPRETING EFFICACY VERSUS EFFECTIVENESS TRIALS

Soil-transmitted helminths are among the world’s most common infections, affecting more than 25% of the world’s population.^4^ Children are most affected by severe infections that can have detrimental long-term effects, including stunting.^5^ Anthelmintic therapy (deworming) is administered to more than 100 million children annually, often in mass drug administration (MDA) programs, and there is some evidence to suggest a potential linear growth benefit from deworming.^6^ However, results from a recent Cochrane review of randomized trials assessing the growth benefits of deworming interventions are mixed. Although some individual trials demonstrate beneficial effects of deworming on childhood growth, the results of pooled analyses demonstrate no statistically significant effects (mean difference −0.02 cm, 95% confidence interval [CI]: −0.17, 0.12).^6^ Interpreting and applying these results requires a careful understanding of the distinction between efficacy and effectiveness in the context of clinical trial design.

Efficacy trials assess how well an intervention can work under ideal circumstances. To determine the potential efficacy of deworming interventions, initial trials need to be conducted among children who are known to have helminth infection at baseline, to assess the effect of treatment in an infected population. Among the 42 trials in children included in the Cochrane review, few trials (2, *N* = 136) were conducted under these conditions and measured linear growth as an outcome (mean difference: 0.10 cm, 95% CI: −0.15, 0.45). Best available estimates of the efficacy of deworming should rely on studies that first screen for helminth infection and treat only those who are infected.^6^

Effectiveness trials, also known as pragmatic trials, assess an intervention’s effect in real world conditions and can be challenging to interpret. Effectiveness trials of deworming use MDA to treat populations independent of individual helminth infection status because it is less expensive and more readily delivered than implementing a screen and treat program. Thus, the measured effectiveness of MDA programs, the most common deworming intervention, to promote linear growth, depends on the prevalence of helminth infection in the population as any improvement in growth would only be expected to occur in children infected with helminths. Conversely, uninfected individuals are not likely to benefit from deworming and will attenuate the population effect size observed in that study. As a result, studies testing MDA deworming interventions conducted in high prevalence settings demonstrate greater growth benefit than studies in lower prevalence populations (mean difference: 0.25 cm, 95% CI: −0.10, 0.60 versus −0.26 cm, 95% CI: −0.74, 0.21).^6^ Although the true effect of deworming any single child infected with helminths may be fixed, the population level effects will depend on the prevalence of infection. As a result, estimates of the population benefit expected if anthelmintics administered by MDA are included in a package targeting stunting must take into account 1) the benefit expected among infected children and how that benefit is likely to translate to population level growth outcomes and 2) interaction effects of package contents.

## MULTIPLE MICRONUTRIENTS: UNDERSTANDING AND EVALUATING THE COMBINED EFFECTS OF INTERVENTIONS

Results from trials of childhood multiple micronutrient interventions provide insight into the potential multiplicative or additive benefits provided by combining interventions into a package. However, although the methodological principles of evaluating the combined effects of interventions are well established, determining how combined interventions interact is complex.^7,8^ The chief aim of trialing multiple interventions is assessing whether the combination offers an added benefit over the single intervention, often referred to as additive effects. Additive effects assume that if treatment A has an effect *E*_A_ over a control and treatment B has an effect *E*_B_ over that same control, then the combination of A and B would have an effect *E*_A_ *+ E*_B_.^9^ Additive effects assume that the interventions work via independent pathways. For example, it would be reasonable to assume that a handwashing intervention and a nutritional intervention do not use the same pathway and therefore, should not interact. However, many interventions have complex or uncertain mechanisms of action, and so it is advisable to test for interactions between combined interventions using factorial randomized trials. Guidance for the assumptions of additive effects have been previously reported.^9^ In rare circumstances, interactions can lead to a greater-than-expected treatment effect (i.e., *E*_AB_ *> E*_A_ *+ E*_B_), referred to as synergy. When an interaction leads to a treatment effect less than the sum of the combined treatments (i.e., *E*_AB_ *< E*_A_ *+ E*_B_) it is termed as antagonism. A common cause of antagonistic interactions is class effect. This occurs when interventions using similar pathways have comparable individual effect sizes but negligible additive effects, because the therapeutic utility of the shared pathway is already fully exploited by a single therapeutic.

Intra-package interactions are extremely relevant in the context of micronutrient interventions which often do not target a single nutrient, but rather try to tackle multiple nutritional deficiencies.^10–12^ In theory, by addressing several stunting-associated deficiencies, childhood micronutrient supplementation should have additive benefits. However, demonstrating additive or synergistic benefit is more challenging than proving a single intervention efficacy.^13^ There may be a maximum threshold of benefit provided by micronutrient supplementation that limits the total linear growth impact achievable. In addition, individual micronutrients may compete for intestinal or hematological transport mechanisms, potentially prohibiting the full effect of each individual intervention.^14–16^

For example, a published meta-analysis of zinc supplementation in children suggests a modest but statistically significant benefit on linear growth, particularly among children who are zinc deficient.^17^ However, results of individual factorial design studies testing zinc in combination with other micronutrients such as vitamin A or iron are inconsistent and do not demonstrate a clear additive benefit.^18–22^ It is possible that the micronutrients exert a class effect or that they compete to exert an effect through a single biological pathway. The difficulty in demonstrating the biological benefit of combining multiple individual micronutrients into a single treatment reinforces the need for careful consideration of potential interactions between constituent interventions of a package when designing any growth promotion package.

To directly extract efficacy and effectiveness knowledge and quantitatively delineate the complex interaction between proximal and distal determinants on growth outcomes, a large knowledgebase combining novel modeling techniques beyond systematic review, meta-analysis, and factorial randomized trials may be helpful to increase learning and knowledge generation.

## DISCUSSION

Anthelmintics and multiple micronutrient interventions serve as examples to highlight the complexity of assessing interventions targeting linear growth and of determining optimal combinations of interventions to achieve maximum potential benefit. Many interventions have not been evaluated for growth benefit in well-designed efficacy trials, and many trials of combined interventions were not designed to evaluate additive effects or antagonism. In addition, the diversity of interventions suggested for inclusion in a possible package targeting linear growth promotion may require delivery through a combination of delivery platforms. Therefore, recommending an optimal linear growth promotion package based on the currently available published literature with any certainty of its beneficial effect is challenging. This, in turn, limits the ability of policymakers to set guidelines for packages of interventions. Single intervention-based recommendations may be a missed opportunity to optimize childhood health.

It may be possible to use novel modeling techniques to integrate existing datasets and find commonalities that are not apparent through systematic review and meta-analysis. However, although using meta-analysis to pool or compare results from the diverse fields of research contributing to the stunting discourse may not always be methodologically appropriate, it may be possible to use nontraditional methods specifically designed to test the integration of different interventions. For example, nonlinear effect models, Markov models, piecewise linear models, principal components models, and machine-learning decision models can be used to evaluate longitudinal growth outcomes in pooled data and explore alternative treatment strategies via clinical trial simulation.

The Healthy Birth, Growth, and Development knowledge integration initiative at the Bill & Melinda Gates foundation is using a variety of analytic methods to model longitudinal growth outcomes and test the effect of interventions using clinical trial data.^23^ Select examples of models developed through this initiative are summarized in Table 1.^24^ Although a careful understanding of efficacy versus effectiveness and potential interactions in the current literature is still required, a combination of these methods applied to pooled datasets could offer new insight into the interactions between, and importance of, growth-promoting interventions. Ultimately, these innovative techniques could be used to inform the design of growth-package interventions before clinical trial testing.

**Table 1:** Examples of models developed for longitudinal growth outcomes and clinical trial simulation through HBGDki initiative^24^

Model name	Description
Full random effects model	Parametric nonlinear model to describe standardized growth (height-for-age *z*-score [HAZ])
Joint distribution of length, weight, and head circumference	Joint parametric nonlinear using nonlinear deceleration structural model
Linear models ordered categorical model for HAZ	Ordered categorical model for HAZ with category probabilities depending on age and other predictors
Multistate Markov model to describe longitudinal changes in HAZ	Multistate model allowing transitions between HAZ categories; modeling-ordered categorical outcomes
Piecewise linear model to describe longitudinal HAZ measures	Piecewise linear growth over specified age intervals. Child-specific birth size and slopes are usually included in the model
Nonlinear mixed effects (NLME) model	Parametric models for pre- and postnatal growth
Bayesian NLME model	Bayesian parametric models for pre- and postnatal growth
Functional principal components model to describe longitudinal measures	Semiparametric model to describe growth
Superimposition by translation and rotation model	NLME model for weight and length/height
Machine-learning models	Ensemble of 1,000 gradient-boosted decision trees

HBGDki = Health Birth, Growth, and Development knowledge integration.

It is clear that high-quality data from interventional trials are needed to inform the development of optimal intervention packages to improve growth in children living in resource-limited settings. However, using systematic review and meta-analysis of the current evidence base to design intervention packages is likely to expend valuable resources testing packages of interventions that are not optimized. New approaches to designing linear growth promotion packages are required.
